# Identification of Potential Therapeutic Agents for Primary Amebic Meningoencephalitis Using Text Mining and Bioinformatics Analyses

**DOI:** 10.1155/ancp/5577830

**Published:** 2026-03-09

**Authors:** Eun Jung Sohn, Hyeonseo Jo, Kun Taek Park

**Affiliations:** ^1^ Department of Biotechnology, Inje University, Gimhae, 50834, Republic of Korea, inje.ac.kr; ^2^ Department of SmartBio, Kyungsung University, Busan, 48434, Republic of Korea, ks.ac.kr

**Keywords:** Gene Ontology, PanDrugs, primary amebic meningoencephalitis, text mining

## Abstract

*Naegleria fowleri*, the brain‐eating ameba, causes primary amebic meningoencephalitis (PAM), a fatal infectious disease that affects the central nervous system (CNS). We aimed to evaluate the functions and potential drugs targeting PAM using text mining and bioinformatics analyses. PAM‐associated genes were identified using a disease database and mined from literature. To identify candidate drugs targeting PAM, 218 genes were analyzed using PanDrugs, drug Manually Annotated Targets and Drugs Online Resource (MATADOR), and the drug Comparative Toxicogenomics Database (CTD) by text mining. Kyoto Encyclopedia of Genes and Genomes (KEGG) and Gene Ontology (GO) functional analyses were performed to examine the mechanism of action of PAM. The GO functions of genes involved in PAM identified by text mining were leukocyte differentiation and the regulation of cytokine production. Disease‐related PAM analyses indicated association with Zellweger syndrome, peroxisomal disease, periodontitis, and leishmaniasis. KEGG enrichment included pathways related to inflammatory bowel disease, malaria, interleukin (IL)‐17 signaling pathway, *Yersinia* infection, Chagas disease, amebiasis, rheumatoid arthritis, pathogenic *Escherichia coli* infection, lipids, atherosclerosis, and peroxisomes. In addition, arsenic trioxide, bortezomib, dasatinib, bosutinib, bevacizumab, paclitaxel, midostaurin, tamoxifen, copanlisib, and pazopanib were identified as potential drugs targeting PAM using PanDrugs software. Our analyses revealed that text mining‐related PAM genes were enriched in several pathways, such as peroxisomes and protein localization. We suggest that PAM is linked to other diseases, such as Zellweger’s syndrome, leishmaniasis, and periodontitis, and provide potential drugs for effective treatment.

## 1. Introduction

*Naegleria fowleri*, a free‐living ameba, is found mainly in soil and freshwater environments that causes primary amebic meningoencephalitis (PAM), a sporadic rare infectious disease of the central nervous system (CNS) [1, 2]. PAM is an acute and progressive disease that develops after a short incubation period. According to the Centers for Disease Control and Prevention, the incubation period for PAM is 1–12 days after exposure. Symptoms are usually not visible until 3–7 days after the infection [3]. Nasal aspiration of trophozoites is the principal route of entry of *N. fowleri* into the human body. *N. fowleri* is frequently referred to as a “brain‐eating ameba” as it causes severe encephalitis after infection, which has a mortality rate of > 95% [3].

Genomic and molecular studies on *N. fowleri* have included detection in environmental samples, such as tap water [4]. In addition, extrachromosomal ribosomal DNA [5], mitochondrial genome [6], and virulence‐associated molecules, including extracellular vesicle–associated proteins of *N. fowleri* [7, 8] have been characterized.

Investigating pathogenic mechanisms of PAM involved in immune evasion and tissue damage by *N. fowleri* help us understand the ameba–host relationship [9–11]. Mouse neutrophils react with *N. fowleri* by releasing extracellular traps [9]. *N. fowleri* excretory–secretory proteins include cathepsin B and cathepsin B‐like cysteine proteases [10]. When comparing *N. fowleri* with the wild‐type, nf‐actin‐overexpressing *N. fowleri* displayed higher phagocytic activity and cytotoxicity [11]. In addition, adhesion, hydrolytic enzymes, and phagocytosis are pathogenic methods used by amebae to injure the host [9, 12].

The text mining technique was developed as a potent tool for extracting knowledge and information from the literature, offering hints for repositioning currently available medications and aiding in future effective treatments [13]. New evidence for the possibility of repurposing existing medications was identified by mining published literature, examining other databases, and using additional searches and analytical techniques [14]. Text mining is an appropriate approach for identifying therapeutic targets and for drug discovery [15].

As PAM is a rare disease in humans, there is no standard treatment. Furthermore, the functions of PAM have not been thoroughly investigated. Systems biology approaches have recently been applied to infectious diseases, such as those reported for *Orientia tsutsugamushi* and *Campylobacter concisus* [16], to identify therapeutic targets. Furthermore, natural compound screening of toxin‐associated proteins in the *N. fowleri* Karachi strain has been performed using in silico techniques for antipathogenic drug discovery [17]. A rare case *of N. fowleri* meningoencephalitis detected using a combination of molecular biology and metagenomic sequencing approaches has recently been reported, demonstrating the diagnostic PAM‐associated limitations [18, 19]. To identify new targets and explore novel therapeutic options for PAM, we identified essential PAM‐associated genes using text mining along with bioinformatics analysis to understand functional characteristics and candidate drugs for PAM.

## 2. Materials and Methods

### 2.1. Text Mining

The DISEASES database (https://diseases.jensenlab.org/) was used for text mining to identify genes associated with PAM. DISEASES is an open‐access database for disease‐gene associations mined from the literature (data source: https://compartments.jensenlab.org/Search). We used the search term “primary amebic meningoencephalitis” to identify genes. Gene lists and confidence scores are provided in Supporting Information.

### 2.2. Identification of PAM Hub Genes

To identify hub genes, the maximal clique centrality (MCC) and eccentricity algorithms of the cytoHubba plugins of Cytoscape (v3.9.1) were used to rank the top 10 nodes (hub genes) of PAM. A protein–protein interaction (PPI) network was constructed using the STRING database (confidence score cutoff = 0.7). To determine the molecular complex detection (MCODE) components in functional gene clusters, The Metascape (v3.5) tool (https://metascape.org/) was used to determine the MCODE components of functional gene clusters [20]. The parameters were set as follows: degree cutoff = 2, node score cutoff = 0.2, *k*‐core = 2, and max depth = 100. Clusters with ≥ 3 nodes were retained. Functional enrichment was performed with default Metascape settings false discovery rate (FDR)‐adjusted *p*‐values < 0.05.

### 2.3. Gene Ontology (GO) and Kyoto Encyclopedia of Genes and Genomes (KEGG) Pathway Analyses

KEGG pathway and GO analyses were performed using the web tool Shiny GO (hinyGO 0.76 [sdstate.edu]) to analyze the enrichment of 218 PAM genes from the disorders web tool for text‐mining linked diseases. A Metascape analysis (https://metascape.org/) was performed to enrich the biological pathways of the 218 PAM genes. Functional enrichment analysis was conducted using the ShinyGO software (v0.76). GO, KEGG, and drug enrichment analyses were performed using FDR‐adjusted *p*‐values < 0.05. Multiple testing correction was applied using the Benjamini–Hochberg method, and terms with a FDR < 0.05 were considered significant.

### 2.4. Enrichment Analyses for Associated Diseases

Using Metascape (https://metascape.org), 218 PAM genes identified by text mining were analyzed using DisGeNET [21]. DisGeNET is a platform for studying the genetic causes of human disorders [22, 23].

### 2.5. Potential Drug Identification for Treating PAM

Shiny GO (ShinyGO 0.76 [sdstate.edu] was used to analyze 218 PAM genes using the Comparative Toxicogenomics Database (CTD) [24] and Manually Annotated Targets and Drugs Online Resource (MATADOR). PanDrugs offers a bioinformatics platform to generate a list of potential drug targets and targetable genes based on genetic variations and unregulated genes identified via genomic analysis (http://www.pandrugs.org). Direct targets, biomarkers, and pathway members were the three categories of the selected target genes [25].

## 3. Results

### 3.1. Text Mining, GO, and Pathway Enrichment Analysis

**Figure 1:**
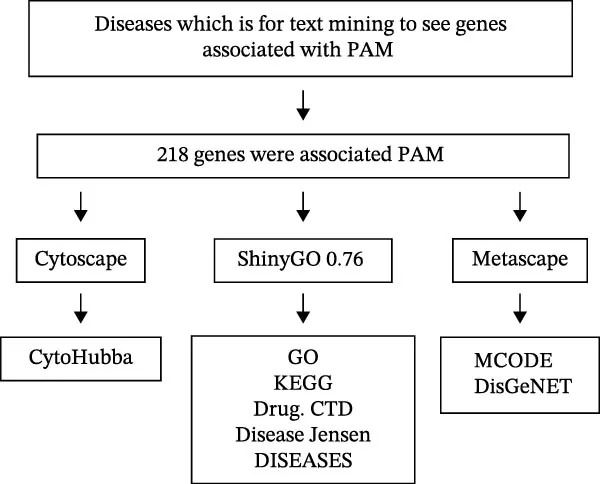
Workflow chart of bioinformatics analysis. GO, Gene Ontology; KEGG, Kyoto Encyclopedia of Genes and Genomes; MCODE, molecular complex detection; PAM, primary amebic meningoencephalitis (https://diseases.jensenlab.org/; accessed January 2024).

DISEASES, a web tool for text mining associated with diseases, was used to identify genes associated with PAM. Referring to the text mining analysis, 218 genes related to PAM were identified and are outlined in a schematic flow chart (Figure 1).

The online tool ShinyGO 0.76 (http://bioinformatics.sdstate.edu/go/) was used to perform GO and KEGG enrichment of the 218 genes. Several enrichment terms were identified, and the top nine significant enrichment terms for biological processes (BPs), cellular components (CCs), and molecular functions (MFs) are presented in Figure 2. BP annotation included transmembrane protein import into intracellular organelles, intracellular protein transmembrane transport, protein transmembrane transport, protein targeting to the mitochondria, establishing protein localization into the mitochondria, protein targeting, protein import, protein transport, and protein establishment (Figure 2A). The most enriched CC annotation was the mitochondrial intermembrane space protein transporter complex, followed by an integral component of the peroxisomal membrane, translocase of the inner mitochondrial membrane 23 (TIM23), and the mitochondrial import inner membrane translocase complex (Figure 2B). Cannabinoid receptor and microfilament motor activity accumulated in the MF category (Figure 2C).

Figure 2Top nine enrichment Gene Ontology (GO) terms and pathways of differentially expressed 281 genes related to PAM from text mining. (A) Biological process (BP), (B) cellular component (CC), and (C) molecular function (MF) related genes for PAM. BP, CC, and MF of GO analysis were performed using ShinyGO 0.77 (http://bioinformatics.sdstate.edu/go/, accessed on January 2024). FDR for DEG with a cutoff value of 0.05.

**Figure:**
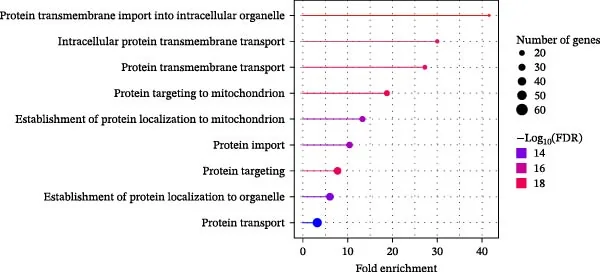
(A)

**Figure:**
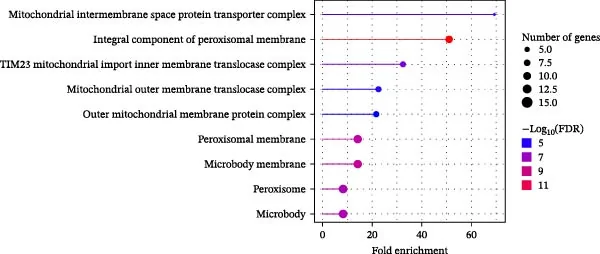
(B)

**Figure:**
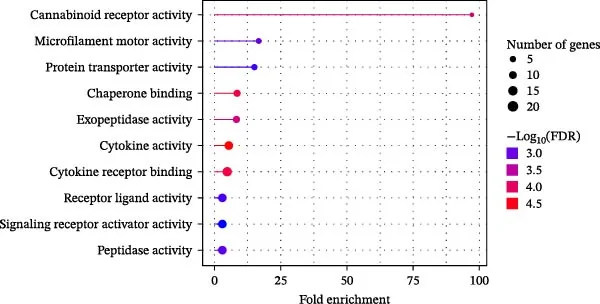
(C)

The network of enriched KEGG was shown in inflammatory bowel disease, malaria, interleukin (IL)‐17 signaling pathway, *Yersinia* infection, Chagas disease, amebiasis, rheumatoid arthritis, pathogenic *Escherichia coli* infection, lipids, atherosclerosis, and peroxisomes (Figure 3A).

Figure 3The Kyoto Encyclopedia of Genes and Genomes (KEGG) and functional analysis of 281 genes related to PAM from text mining. (A) KEGG pathway analysis of differentially expressed genes (DEGs). The KEGG analysis was performed using ShinyGO 0.77 (http://bioinformatics.sdstate.edu/go/). The green nodes represent an enriched pathway, and their size is associated with a group of larger genes. Overlapping genes are represented by thicker borders. If two nodes share at least 20% of a gene, they are linked (http://bioinformatics.sdstate.edu/go/, accessed on January 2024). FDR‐adjusted *p*‐values < 0.05 were considered significant. The nodes illustrate the enriched BP, and the edges show the interaction among them. The thicker the edges, the more genes overlap. The intensity of color represents the significance of the interaction. (B) Bar graph showing the top enriched pathways and GO biological processes. The biological functions of hub genes were analyzed via the Metascape database. (https://metascape.org/, accessed on January 2024). FDR‐adjusted *p*‐values <0.05.

**Figure:**
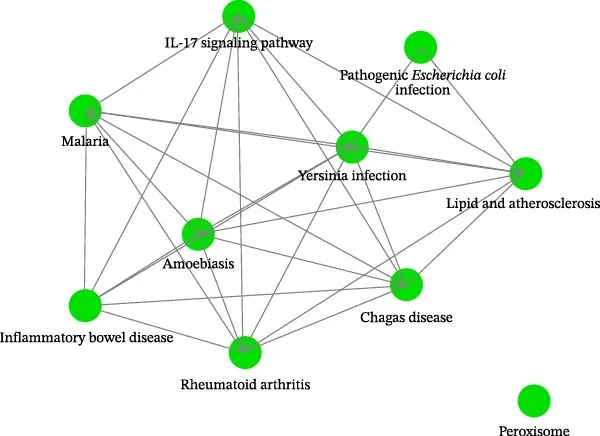
(A)

**Figure:**
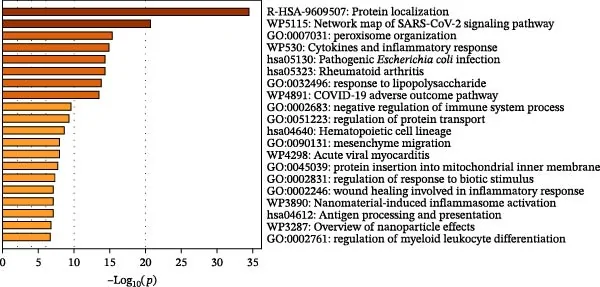
(B)

Using Metascape, enrichment analysis indicated R‐HAS‐9609507 protein localization, a WP5115 network map of severe acute respiratory syndrome coronavirus 2 (SARS‐CoV‐2) signaling pathway, and GO0007031 peroxisome organization (Figure 3B).

### 3.2. Screening of Hub Genes and PAM Modules

To identify the hub genes of PAM, the PPI networks for 218 PAM genes obtained from the text‐mining dataset were constructed using the Cytoscape and cytoHubba plugins. Ten hub genes from two 218 genes of PAM obtained by text mining dataset were included, glyceraldehyde‐3‐phosphate dehydrogenase (GAPDH), toll‐like receptor (TLR) 4, IL‐1 beta (IL‐1B), IL‐10, cluster of differentiation (CD) 4, tumor necrosis factor (TNF), IL‐6, IL‐2, IL‐4, and C–C motif chemokine ligand (CCL)2 by MCC (Figure 4A, left) and GAPDH, Jun proto‐oncogene, AP‐1 transcription factor subunit (JUN), albumin (ALB), glutamyl aminopeptidase (ENPEP), DNA topoisomerase (TOP) 1, Jun dimerization protein (JDP) 2, GABA type A receptor–associated protein like (GABARAPL) 2, caspase (CASP) 3, CASP 1, and CCL5 by eccentricity (Figure 4A, right). The top five hub genes identified by the cytoHubba plugin employing MCC and eccentricity algorithms were GAPDH, TNF, IL‐1B, IL‐10, and CD4 by MCC analysis, and a distinct set of top hub genes, including GAPDH, JUN, ALB, CASP3, and CASP1 by eccentricity analysis.

Figure 4Hub gene screening of differentially expressed genes (DEGs) and molecular complex detection (MCODE) analysis of 281 genes related to PAM from text mining. (A) Ten hub genes were identified by maximal clique centrality and eccentricity algorithms in cytoHubba. Deeper red colors indicate higher algorithm scores and more critical targets. (B) MCODE analysis for PAM from text mining. PAM‐related genes were classified into seven modules based on their activities and protein–protein interaction networks. MCODE analysis using Metascape was performed for 281 genes related to PAM from text mining (https://metascape.org/, accessed on January 2024). FDR‐adjusted *p*‐values <0.05.

**Figure:**
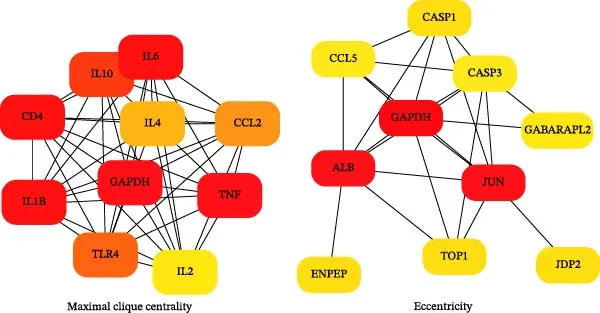
(A)

**Figure:**
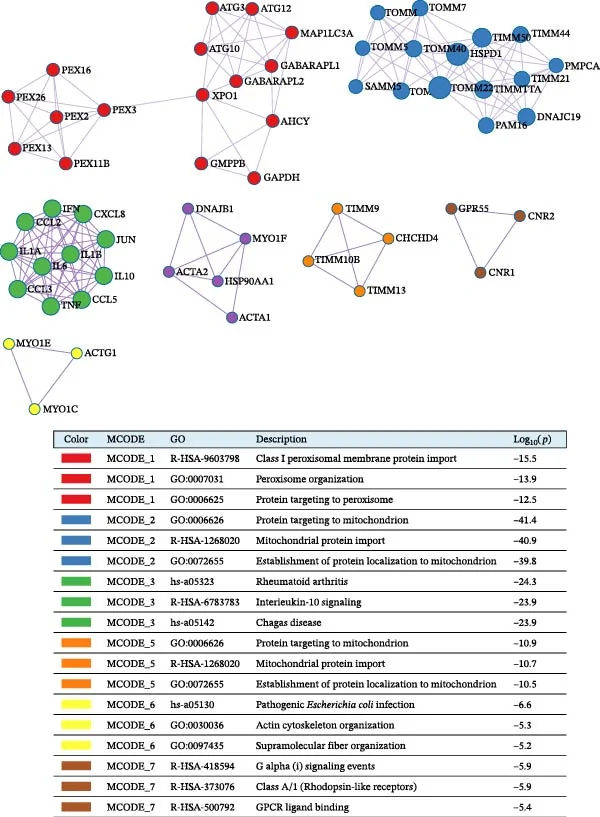
(B)

Module analysis using the Metascape tool was performed to identify the functional clusters of 218 PAM genes. In the MCODE analysis using Metascape, 218 PAM genes from the text mining dataset were grouped into six modules. MCODE analysis revealed that the DEGs were classified into seven modules. The pathways in module 1 were associated with class I peroxisomal membrane protein import, peroxisome organization, and protein targeting of peroxisomes. Module 2 included proteins targeting the mitochondria, mitochondrial protein import, and the establishment of protein localization to the mitochondrion in module 2. Rheumatoid arthritis, IL‐10 signaling, and Chagas disease were enriched in Module 3 (Figure 4B). Functional clustering via Metascape MCODE analysis grouped these genes into six modules: module 1 was associated with peroxisome organization, module 2 with mitochondrial protein targeting, and module 3 with immune‐related pathways, including IL10 signaling and rheumatoid arthritis.

### 3.3. Novel Targeted Drug Prediction for PAM

To identify candidate drugs targeting PAM, 181 target genes selected by text mining were used in PanDrugs to describe drug interactions, drug status, and potential drug responses. The PAM drug family includes oxidoreductases, rhodopsin family, hydrolases, estrogen‐like receptors, cytokines, monoclonal antibodies, serine/threonine kinases, voltage‐gated ion channels, receptor tyrosine kinases, and vascular endothelial growth factor receptor (VEGFR) inhibitors (Figure 5A). Drug prediction for PAM revealed several potential novel drugs and genes in Figure 5B. Genes, such as CASP 3, heat shock protein 90 alpha family class A member 1 (HSP90AA1), surfactant protein A4 (SPA4), IL‐6, Jun proto‐oncogene, AP‐1 transcription factor subunit (JUN), transducin beta‐like 1X‐linked (TBL1X), and TNF showed potential targets of arsenic tridoxide. family tyrosine kinase (FGR) proto‐oncogene, Src FGR, HSP90AA1, HSP70, HSPA4, proteasome subunit alpha type‐4 (PSMA4), and TLR4 were involved in potential targets of bortezomib (Figure 5B and Supporting Information 1: Table S1).

Figure 5Identification of potential therapeutic targets and ranked drugs for PAM through PanDrugs‐based text mining. (A) The drugs family for PAM using Pan drugs. (B) The potential druggable target and drugs for PAM using PanDrugs. Pandrugs (http://www.pandrugs.org) was carried out for 281 genes related to PAM from text mining. (http://www.pandrugs.org/, accessed on January 2024).

**Figure:**
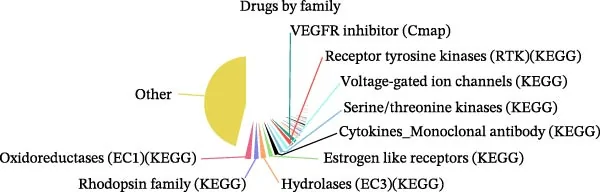
(A)

**Figure:**
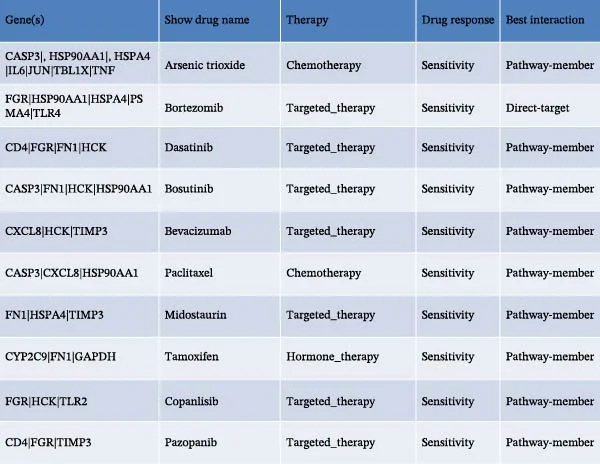
(B)

Drug MATADOR and drug CTD from the ShinyGO 0.76 online tool were used to identify potential drug–target relationships. Drug MATADOR showed that the top 10 drugs associated with PAM were budesonide, linomide, polyinosinic polytidylic acid, imiquimod, sulfasalazine, thalidomide, fluticasone propionate, and troglitazone (Figure 6A). Drug CTD demonstrated that the top 10 enrichment chemicals or drugs were hemagglutinins, silymarin, bromoindirubin‐3^′^oxime, E804 bisindole, lipopolysaccharide *E. coli* O111B4, quartz, ionomycin, sodium dichromate, SB203580, and vehicle emissions (Figure 6B).

Figure 6Potential therapeutic drugs from 281 genes related to PAM from text mining. The top list of potential drugs for PAM using the drug MATADOR (A) and drug CTD (B). Drug MATADOR and drug CTD were carried out for 281 genes related to PAM from text mining. Drug MATADOR and drug CTD resulted from ShinyGO 0.77 (http://bioinformatics.sdstate.edu/go/, accessed on January 2024). FDR‐adjusted *p*‐values <0.05.

**Figure:**
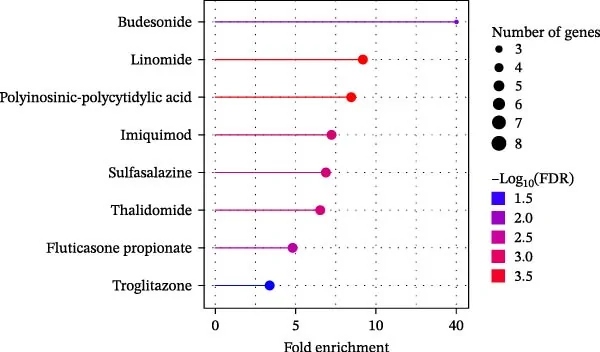
(A)

**Figure:**
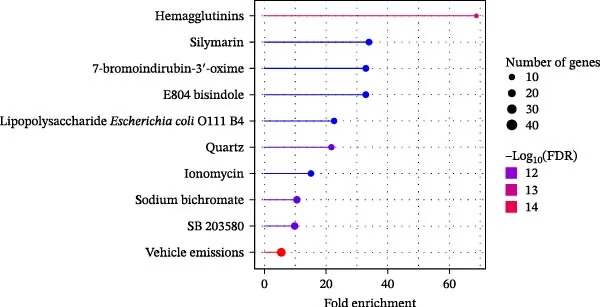
(B)

### 3.4. DisGeNET of 216 Genes From PAM

To identify disease‐related genes in PAM, Jensen DISEASES provided Shiny GO 0.76 online, and DisGeNET from Metascape was performed. Jensen DISEASES from Shiny GO 0.76 online demonstrated that 216 PAM genes were involved in Zellweger syndrome, peroxisomal disease, leishmaniasis, meningitis, uveitis, candidiasis, influenza, lung disease, and arthritis (Figure 7A). DisGeNET database disease enrichment analysis using the Metascape tool was associated with leishmaniasis, visceral and chronic periodontitis, encephalitis, endotoxemia, toxoplasmosis, and leishmaniasis (Figure 7B).

Figure 7Enrichment analysis of diseases for PAM. (A) The top 10 list of Jensen DISEASES for PAM. PAM‐related genes (281) from text mining were performed using ShinyGO 0.77 (http://bioinformatics.sdstate.edu/go/) to identify Jensen DISEASES. (B) The top 10 list of DisGeNET. DiGeNET for 281 genes related to PAM from text mining resulted from the Metascape (https://metascape.org/, accessed on January 2024). FDR‐adjusted *p*‐values <0.05.

**Figure:**
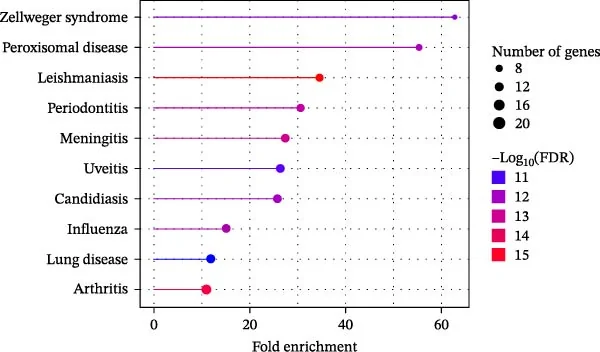
(A)

**Figure:**
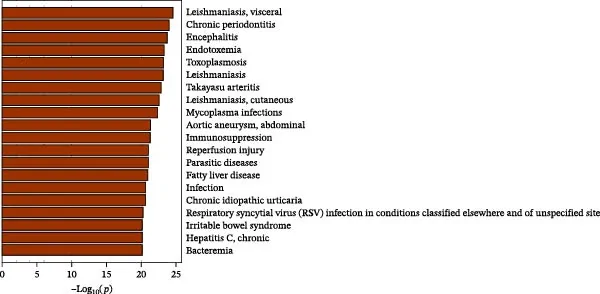
(B)

### 3.5. Functional Comparison of PAM, Leishmaniasis, and Periodontitis Infections in Cells

Figure 7 shows that PAM is associated with leishmaniasis and periodontitis. To understand the relationship between PAM and leishmaniasis and periodontitis, a text mining web tool was used to identify genes involved in leishmaniasis and periodontitis. A Venn diagram was used to identify the genes related to PAM, leishmaniasis, and periodontitis. The diagram represented the 38 common targets of PAM, leishmaniasis, and periodontitis (Figure 8A).

Figure 8Enrichment analysis of common genes by text mining among amebic meningoencephalitis, leishmaniasis, and periodontitis. (A) A Venn diagram of the intersection of genes by text mining among amebic meningoencephalitis, leishmania, and periodontitis. The biological process of Gene Ontology (https://diseases.jensenlab.org/ accessed on January 2024). (B) Kyoto Encyclopedia of Genes and Genomes pathways. (C) Enrichment analyses of the intersection of genes from amebic meningoencephalitis, leishmaniasis, and periodontitis by text mining (http://bioinformatics.sdstate.edu/go/, accessed on January 2024). FDR‐adjusted *p*‐values <0.05.

**Figure:**
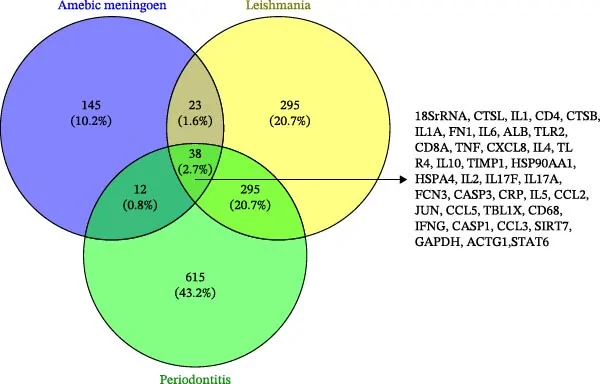
(A)

**Figure:**
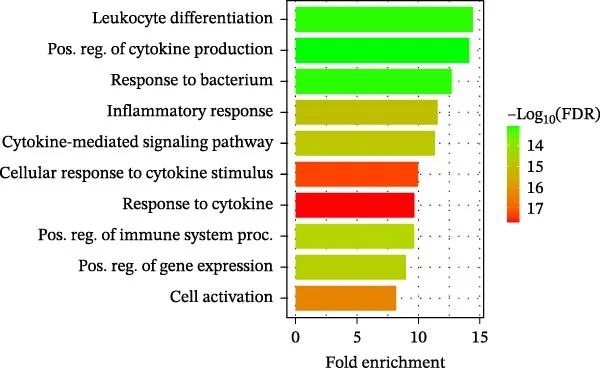
(B)

**Figure:**
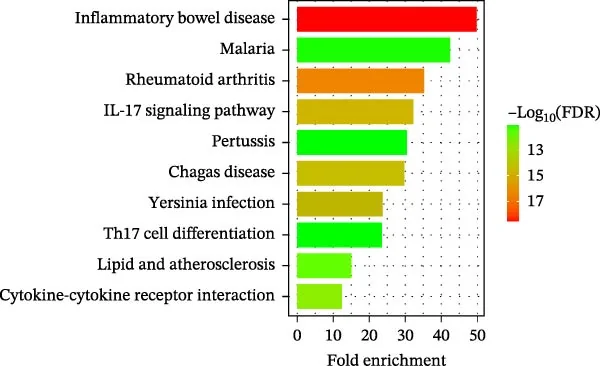
(C)

The BP of GO analysis of 38 common genes for the three diseases (PAM, leishmaniasis, and periodontitis) revealed that these genes were related to leukocyte differentiation, positive regulation of cytokine production, response to the bacterium, inflammatory response, cytokine‐mediated signaling pathway, response to cytokine, cellular response to cytokine stimulus, positive regulation of immune system processes, positive regulation of gene expression, and cell activation (Figure 8B). KEGG pathway involving 32 genes included inflammatory bowel disease, malaria, rheumatoid arthritis, IL‐17 signaling pathway, pertussis, Chagas disease, *Yersinia* infection, Th17 cell differentiation, lipid and atherosclerosis, and cytokine–cytokine receptor interaction (Figure 8C).

## 4. Discussion

PAM is a rare disease, with the highest mortality rate among infectious diseases [26–28]. There are only 11 PAM survivors out of 260 cases worldwide [29–31]. Although the pathogenesis of *N. fowleri* has been investigated over the past 50 years, the pathways and pathogenic mechanisms of human infection with *N. fowleri* remain unknown. In this study, our analyses revealed that genes related to PAM from text mining were enriched in several pathways, such as peroxisomes and protein localization. We suggest that PAM is linked to inflammatory diseases, such as Zellweger syndrome, leishmaniasis, and periodontitis, providing potential drugs for PAM.

Several metabolic processes are involved in peroxisomes and other important subcellular organelles in mammals [32]. Primary peroxisomal functions include lipid peroxidation and beta‐oxidation of very‐long‐chain fatty acids [33]. Peroxisomes are linked to various human diseases including inflammation, abnormal CNS morphology, and diabetes [34–36]. A deadly condition known as Zellweger syndrome is the absence of functional peroxisomes [37]. Patients with Zellweger syndrome experience profound neurological abnormalities, such as dysfunction or demyelination, defects in neuronal migration, neonatal hypotonia, and craniofacial dysmorphism [38]. In this study, GO and KEGG pathway analyses of PAM genes included peroxisomes. Additionally, the Jensen DISEASES analysis showed that PAM was associated with Zellweger syndrome. Therefore, peroxisomes may be an essential pathway in PAM.

In our study, the top five hub genes, such as *GAPDH*, *TNF*, *IL-1B*, *IL-10*, and *CD4*, were associated with neuroimmune processes. GADH mediates neuroinflammatory signaling and neuronal stress responses by influencing cytokine expression and microglial function [39]. TNF‐α plays a role in CNS homeostasis and neuroinflammation [40]. IL‐1B mediates microglial activation and pro‐inflammatory signaling, whereas IL‐10 exerts anti‐inflammatory effects to maintain CNS immune homeostasis [41]. CD4^+^ cells mediate adaptive immune responses influencing neuroinflammation [42]. Functional clustering via Metascape MCODE analysis revealed modules linked to peroxisomal and mitochondrial processes (modules 1 and 2) and immune‐related pathways including IL‐10 signaling (module 3), suggesting that metabolic regulation intersects with immune signaling in PAM. These findings suggest potential molecular mechanisms of neuroimmune dysregulation in PAM.

Periodontitis is a constant source of infection in conditions, such as peripheral artery disease, cardiovascular disease, and rheumatoid arthritis. It poses the risk of mortality and morbidity associated with osteoporosis, diabetes, and insulin resistance [43–45]. Leishmaniasis is a neglected tropical illness in most tropical and subtropical regions worldwide [46, 47]. In this study, the DisGeNET and Jensen’s disease approaches presented a potential connection between PAM, leishmaniasis, and periodontitis. Specifically, this study showed that genes from the text mining of PAM, leishmaniasis, and periodontitis were associated with leukocyte differentiation, inflammatory responses, and cytokine responses. This association may provide essential clues for identifying common molecular targets for monitoring PAM diseases.

PAM drug discovery techniques are constantly used to investigate its potential for repurposing. Azithromycin, amphotericin B, miltefosine, fluconazole, and rifampicin are the current medications for treating PAM, based on the recommendations of the Centers for Disease Control and Prevention [48]. Miltefosine, the first oral leishmaniasis treatment [49], has been described as a promising therapy for PAM and is the only one that has proven efficacy in treating PAM [50]. In this study, we showed that PanDrugs had several potential targets and therapeutic candidates. In Supporting Information 1: Table S1, miltefosine was one potential therapeutic candidate for PAM by targeting TNF. In addition, IL, CD4, and CASP 3, the top 10 high‐scoring hub genes in PAM by the PPI network, showed potential druggable targets for PAM by PanDrugs. In addition, arsenic trioxide, bortezomib, dasatinib, bosutinib, bevacizumab, paclitaxel, midostaurin, tamoxifen, copanlisib, and pazopanib were potential drugs for the treatment of PAM using the PanDrugs software (Figure 5). Furthermore, CTD and MATADOR demonstrated potential for PAM (Figure 6). Thalidomide is an immunomodulatory drug that is effective against the activation of the TNF pathway in myeloma patients, likely due to MAP2K3 mutation [51]. Imiquimod is an immunosuppressive drug approved for the topical treatment of superficial basal cell carcinoma, external genital perianal warts, and actinic keratosis [52]. Thalidomide is well known for its potent anti‐inflammatory property by inhibiting TNF‐α and related pro‐inflammatory cytokines [53]. Excessive cytokine responses, such as TNF‐α, play a critical role in tissue damage during *Entamoeba histolytica* infection [54]. Therefore, although thalidomide does not show a direct amebicidal effect, its ability to modulate the expression of inflammatory cytokines may indirectly reduce immune‐mediated tissue damage. In addition, thalidomide and its analogs, such as lenalidomide and pomalidomide, have demonstrated neuroprotective function by suppressing neuroinflammation, regulating microglial activation, and reducing oxidative stress [55, 56]. Similarly, imiquimod has not been reported to have direct anti‐amebic effects but is known as an immune‐stimulatory agent via TLR 7 activation, which may enhance host immune responses against infection [57]. Taken together, our findings indicate that the potential benefits of these compounds in amebic infections are likely mediated through indirect immunological mechanisms rather than direct antiparasitic activity.

## 5. Conclusions

This study improves our understanding of the functional pathways of PAM and suggests potential drugs for treating PAM. In this study, we identified a new set of genes involved in key signaling and functional pathways in PAM. In addition, we demonstrated that PAM might be associated with peroxisomes and may be linked to other diseases, such as leishmaniasis and periodontitis. In this study, in silico analyses were used to identify the links between genes, pathways, and prospective treatments. Therefore, further experimental validation is required. In addition, the limited availability of PAM‐related genes in public databases may limit the scope of the pathways and disease connections. Despite these limitations, this study provided a comprehensive analysis using text mining and bioinformatics to identify potential therapeutic targets and drugs for intractable diseases.

**Nomenclature**
ALB: Albumin
BP: Biological process
CC: Cellular component
CASP: Caspase
CD: Cluster of differentiation
CCL: C‐C motif chemokine ligand; cluster of differentiation
CTD: Comparative Toxicogenomics Database
FDR: False discovery rate
GO: Gene Ontology
Hsp7: Heat shock protein family
A KEGG: Kyoto Encyclopedia of Genes and Genomes
JUN: Jun proto‐oncogene, AP‐1 transcription factor subunit
MATADOR: Manually Annotated Targets and Drugs Online Resource
MCC: The maximal clique centrality
*N. fowleri*: *Naegleria fowleri*
MF: Molecular function
TBL1X: transducin beta‐like 1X‐linked
TLR: Toll‐like receptor
TNF: Tumor necrosis factor
JDP: Jun dimerization protein
PAM: Primary amebic meningoencephalitis
SARS‐CoV‐2: Syndrome coronavirus 2
VEGFR: Vascular endothelial growth factor receptor.

## Author Contributions

Eun Jung Sohn designed the project and carried out experiments. Eun Jung Sohn and Hyeonseo Jo conducted data and visualization analysis. Eun Jung Sohn and Kun Taek Park wrote the manuscript.

## Funding

Following are results of a study on the “Busan Regional Innovation System & Education (RISE)” Project, supported by the Ministry of Education and Busan Metropolitan City.

## Ethics Statement

The authors have nothing to report.

## Conflicts of Interest

The authors declare no conflicts of interest.

## Supporting Information

Additional supporting information can be found online in the Supporting Information section.

## Supporting information


**Supporting Information 1** To find several targets and therapeutic candidates for PMA, PanDrugs (https://pandrugs.org/) was used.

**Supporting Information 2** Gene lists and confidence scores (source https://compartments.jensenlab.org/Search).

## Data Availability

Data will be made available upon request. The data that support the findings of this study are available from the corresponding author upon reasonable request.
